# Can follow-up study questions about correct and consistent condom use reduce respondent over-reporting among groups at high risk? An analysis of datasets from six countries

**DOI:** 10.1186/1742-4755-7-9

**Published:** 2010-06-11

**Authors:** Varja Lipovsek, Kim Longfield, Justin Buszin

**Affiliations:** 1 Population Services International, 1120 19th Street, NW, Suite 600, Washington, DC 20036, USA

## Abstract

### Purpose

This study sought to determine whether including two types of questions in surveys administered via face-to-face interview can contribute to more accurate measures of correct and consistent condom use among populations at risk for HIV. The study compared initial rates of self-reported condom use to rates found when respondents were asked to provide confirmation of correct and consistent use via follow-up questions.

### Methods

Paired t-test analyses were conducted on data from 11 surveys of female sex workers and men who have sex with men, from five Central American countries and the Dominican Republic. All surveys included either a test-retest item for consistent condom use or else a combination of the test-retest item and a second item measuring correct condom use.

### Results

In all 11 datasets, the proportion of respondents qualifying as consistent condom users decreased significantly after answers to either one or both follow-up questions were taken into account. In the six datasets from surveys of female sex workers, the difference between the initial and final level of self-reported condom use ranged from 4.3% to 23.2%. In the five datasets from surveys of men who have sex with men, the difference between the initial and final level of self-reported condom use ranged from 9.9% to 37.0%.

### Conclusion

Given the amount of recall bias and social desirability bias usually associated with condom use survey items, a measure that identifies a lower proportion of condom users than initially found is taken to be more accurate. The two follow-up questions examined in this study appear to substantially reduce the proportion of people claiming consistent condom use. As most behavioral surveys rely on self-reported measures, the addition of such questions could significantly improve estimates of consistent condom use. We therefore recommend that these and other types of follow-up items be added to future condom use surveys and evaluated further as potential means of obtaining more accurate information about this important behavior.

## Background

Worldwide, sexual contact is the most common route of HIV transmission among general populations, as well as among specific subgroups such as commercial sex workers and men who have sex with men [1]. Condom use therefore remains central to HIV prevention interventions, and behavioral surveillance surveys as well as programmatic evaluations typically focus on self-reported condom use as an important indicator. However, these measures, like other self-reported behavioral measures, have always been problematic; one of the most common issues is thought to be over-reporting of condom use as a result of recall bias or social desirability bias [2-8]. Researchers have published a number of reviews on the challenges of accurately measuring sexual behavior in the past decade, and have issued calls for improving and standardizing measures and methods [5,9-13]. The general consensus is that the reliability and validity of self-reporting is questionable; but there is also agreement that it is, by and large, the only feasible way to measure sexual behaviors.

There has also been much debate about which methods are best suited for eliciting such information. Recent evidence indicates that while self-administered instruments such as computer-based surveys appear to often yield higher and more plausible estimates of sexual risk behavior, this superiority may well be compromised by the potential for the respondent to not understand questions. This could result in higher item non-response as well as skewed responses [11,12]. In most settings, with the possible exception of highly literate groups, some version of face-to-face interviewing appears to still be the optimal way to gather condom use data.

A number of strategies have been suggested to reduce over-reporting of condom use in face-to-face interviews. For example, some studies have sought to combine behavioral surveys with assessments of biological markers such as the presence of sexually transmitted infections (STIs), counting only self-reported condom users who do not present with STI symptoms as consistent condom users [14,15]. Although informative, this practice is financially and practically unfeasible in many research settings. Instead, current practice in measuring self-reported behaviors favors becoming more specific in the phrasing of behavioral questions, particularly when asking about consistent condom use over a period of time, and about correct condom use [12,13,16].

Recall bias and social desirability bias still remain as potential problems, as does the matter of how survey items are interpreted by respondents. The phrasing of questions about "consistent condom use" and "correct condom use" may lead people to erroneously over-report both behaviors. Many problems appear to result from ambiguity in the questions rather than the intentional or unintentional misreporting of condom use [13,17] Since most structured questionnaires prohibit an in-depth discussion of the meaning of questions and terms, a series of "quality checks" can be built into the questionnaire. Such quality checks generally include the following six strategies: (1) asking specifically about different types of partners - e.g., regular, casual, or commercial [13,16]; (2) asking specifically about types of sexual intercourse - e.g., vaginal or anal [13]; (3) asking about coital frequency and condom use based on this frequency [12,13,16]; (4) limiting the recall period to no more than three months (with some exceptions for groups with relatively infrequent sexual activity, such as adolescents) [13,18]; (5) specifying correct condom use and asking about condom failure [13,14,19,20]; (6) including test-retest items [5,11,13]. These strategies are of particular relevance for interventions which aim to increase condom use, because inflated measures can result in inaccurate benchmarks set for program achievement, or in a decline in condom promotion activities because planners believe that a high level of behavior change has already taken place.

This paper reports on an investigation of whether two questionnaire items which specify correct condom use and employ a particular version of the test-retest approach regarding consistent condom use contribute to more accurate measures of condom use among populations at risk for HIV. The study compared initial rates of self-reported condom use to rates found when respondents were asked to provide confirmation of correct and consistent use via two follow-up questions. It is hoped that our findings will encourage the adoption of such items in future research that relies on self-reported condom use measures.

## Methods

No primary data were collected for this study. Analyses were performed using existing datasets from behavioral surveys carried out in 2007 and 2008 by Population Services International (PSI), a non-profit global health organization. Datasets were included if they met the following four criteria: (1) the survey population belonged to a group considered at high risk for HIV; (2) survey participants were selected using probability-based sampling; (3) the survey measured coital frequency and condom use based on this frequency; and (4) the survey included either a test-retest item for consistent condom use or else a combination of the test-retest item and a second item measuring correct condom use. Surveys from El Salvador, Guatemala, Honduras, Nicaragua, Panama, and the Dominican Republic yielded datasets that were suitable for inclusion in the study. The populations at high risk targeted in those surveys were female sex workers and men who have sex with men. Ethical approval was obtained for each individual study prior to data collection.

Study populations were either female commercial sex workers or men who have sex with men. In all surveys of female commercial sex workers, coital frequency was established first for each type of sexual partner, followed by a question about how many of those acts were protected by condoms. Partners were classified as follows: "new" clients were those whom the respondent had met three times or less; "fixed" clients were those the respondent had met more than three times, and "trusted" clients were those who did not necessarily pay for sex. Respondents for whom the number of sexual acts equaled the number of acts in which a condom was used were coded as "initial consistent" users.

Then, the Dominican Republic respondents were asked two follow-up questions for each type of client: "Was there any time in the past 30 days when you could not use a condom with this client?" (i.e., the test-retest item), and, "Did you always use a condom from the beginning to the end of the sexual act with this client?" (i.e., the correct condom use item). Only respondents who confirmed consistent and correct condom use in their answers to both questions were considered to be true consistent users, by type of client. The same two follow-up questions were asked in the surveys administered to female sex workers in the other five countries, but for all clients together. Again, only respondents who confirmed using condoms in their answers to both follow-up questions were considered to be true consistent condom users.

In all of the surveys interviewing men who have sex with men, respondents were first asked to report on the frequency of sex in the past 30 days with three types of partners: occasional, regular, and commercial. They were then asked about the frequency of condom use during those sexual encounters. Respondents were identified as "initial consistent" condom users if the number of sexual acts was the same as the number of sexual acts involving condom use. The follow-up question asked if there was any time in the past 30 days when they did not use a condom with a partner. (The type of partner was not specified.) Only those respondents who answered no to the follow-up question were considered to be true consistent condom users.

The two specific items of interest were the test-retest item on consistent condom use and an item measuring correct condom use. The test-retest question was asked of respondents who reported always using condoms during a specified time period. They were asked to think about the specified time period again, and to confirm that there was no instance in that time period when a condom was not used. "Correct" condom use was defined as "using a condom from beginning to end of intercourse." This item followed the test-retest item; i.e., respondents who said they were consistent condom users without exception were also asked if they always used a condom from the beginning to end of intercourse.

Commonly, test-retest reliability analysis checks for consistency of answers by repeating the same question at different times in the survey, and accepts as "true" only the responses that are consistent with each other [5,11,13]. The analytical approach employed in this study is somewhat different, as it aims to quantify the variation between initial measures of consistent condom use levels and those measures obtained by asking one or two follow-up survey questions. Therefore, analyses consisted of paired t-tests and were performed on the before-and-after responses to the two items of interest. For surveys that included both the test-retest question about consistent condom use and the "beginning to end" question about correct condom use, the proportion of respondents who initially reported consistent condom use was compared to the proportion of respondents who met the criteria imposed by *both *follow-up questions. For surveys that included only the test-retest question about consistent condom use, the proportion of respondents who initially reported always using condoms was compared to the proportion of respondents who could still be considered consistent condom users on the basis of their response to that follow-up question.

The paired t-test analyses yielded the differences between the before-and-after responses, as well as the standard error of the difference and its statistical significance. Since responses from the same individual in the same survey were compared, there was no need to control for sampling strategy or socio-demographic characteristics.

## Results

Table 1 summarizes the study samples which were drawn from six countries. Studies were comprised of respondents belonging to two groups considered at high risk of HIV: female sex workers and men who have sex with men. Study populations were selected using either time-location cluster sampling or respondent-driven sampling. Sample sizes ranged from 297 to 790 respondents.

**Table 1:** Description of study samples

Country	Year	Target population	Type of sample	Sample size
Dominican Republic	2008	Female commercial sex workers	Multi-stage cluster sampling	790

El Salvador	2007	Female commercial sex workers	Multi-stage cluster sampling	588
	
	2007	Men who have sex with men	Respondent-driven sampling	592

Guatemala	2007	Female commercial sex workers	Multi-stage cluster sampling	520
	
	2007	Men who have sex with men	Respondent-driven sampling	591

Honduras	2007	Female commercial sex workers	Multi-stage cluster sampling	509
	
	2007	Men who have sex with men	Respondent-driven sampling	565

Nicaragua	2007	Female commercial sex workers	Multi-stage cluster sampling	511
	
	2007	Men who have sex with men	Respondent-driven sampling	297

Panama	2007	Female commercial sex workers	Multi-stage cluster sampling	510

	2007	Men who have sex with men	Respondent-driven sampling	596

Table 2 presents the results from paired t-tests for surveys including both the test-retest item on consistent condom use and the item on correct condom use. As the table shows, the proportion of respondents who still could be counted as consistent condom users after the two follow-up questions was significantly lower in all of the samples, by 4.3% to 23.2%. The largest difference was in Panama while the smallest difference was found in Guatemala.

**Table 2:** Paired T-test results for surveys that include both follow-up questions for respondents initially reporting consistent condom use

Respondent group*	Considered consistent condom user on basis of initial response	Considered consistent condom user after both follow-up questions	% difference	SE	Sig (2-tailed)
	%	%			

Dominican Republic-new clients	44.6	35.7	-8.8	0.010	p < .001

Dominican Republic-fixed clients	42.5	32.9	-9.6	0.010	p < .001

Dominican Republic-trusted clients	16.3	12.0	-4.3	0.007	p < .001

El Salvador (all clients)	89.2	71.6	- 17.6	0.016	p < .001

Guatemala (all clients)	57.1	51.3	- 5.8	0.013	p < .001

Honduras (all clients)	84.1	65.1	- 19.0	0.017	p < .001

Nicaragua (all clients)	81.3	65.3	- 16.0	0.016	p < .001

Panama (all clients)	81.1	57.9	- 23.2	0.019	p < .001

* All respondents were female commercial sex workers.

SE: Standard error

Sig.: Significance of the t-test

Table 3 presents the results from paired t-tests for surveys including the test-retest item on consistent condom use. The key difference between Tables 2 and 3 is that in Table 3, only the test-retest item was included (and not the item relating to correct condom use). All study populations represented in Table 3 were men who have sex with men. As the table shows, the proportion of respondents who still could be counted as consistent condom users after the follow-up question was significantly lower in all of the samples, by 9.9% to 37.0%.

**Table 3:** Paired T-test results for surveys that include "no exceptions" follow-up question for respondents initially reporting consistent condom use

Respondent group*	Considered consistent condom user on basis of initial response	Considered consistent condom user after the "no exceptions" follow-up question	% difference	SE	Sig (2-tailed)
	%	%			

El Salvador	73.4	63.5	- 9.9	0.013	p < .001

Guatemala	79.7	65.6	- 14.1	0.015	p < .001

Honduras	77.9	61.5	- 16.4	0.017	p < .001

Nicaragua	68.7	51.5	- 17.2	0.022	p < .001

Panama	89.1	52.1	- 37.0	0.022	p < .001

* All respondents were men who have sex with men.

SE: Standard error

Sig.: Significance of the t-test

## Discussion & Conclusions

The purpose of our study was to explore the practical application of two recommendations from the field of measuring sexual behavior, and to quantify the difference between reported consistent condom use levels when condom use is measured once and when it is measured again via confirmatory questions. The two recommendations are (1) to include test-retest questions regarding consistent condom use, and (2) to include questions regarding the correct use of condoms. Analyses of 11 datasets from six countries showed that in all of the datasets, the proportion of respondents qualifying as consistent condom users decreased significantly after answers to either one or both follow-up questions were taken into account.

The correct condom use question captures an essential component of condom use (i.e., using the condom from beginning to end of intercourse), while the test-retest item on consistent condom use prompts the respondent to affirm that there were no exceptions to a condom having been used consistently. This form of test-retest item may actually be more useful than a simple test-retest item which repeats the same question and checks for internal consistency, because asking about exceptions encourages the respondent to think about the given behavior more carefully, possibly yielding a more accurate answer.

Our investigation contributes to a growing body of research that demonstrates how follow-up items can be used to increase the internal validity of surveys measuring condom use. The particular items employed in this case, a test-retest question and a question about correct condom use, reflect two specific recommendations made by Noar et al on the basis of a review of 56 studies of sexual risk behavior [13]; our findings substantiate those recommendations.

As with all research, this study has limitations. There was no primary data collection for the purposes of this study. The original surveys were designed to develop, monitor, and evaluate condom use interventions: available datasets were mined for the information presented here. Datasets are comprised of female sex workers and men who have sex with men, and results may not be generalizable to other populations. The datasets are limited to Central America and the Dominican Republic, and therefore generalizability to other geographical regions is uncertain as well. Furthermore, it should be noted that the 37.0% difference in the Panama cohort responding to the sole confirmatory question about consistent condom use is much larger than any of the other differences observed in this study. The finding leads us to speculate that for the survey in question there may have been meaningful differences in the sequence in which questions were asked, errors in data entry, or other irregularities in the dataset. Finally, as with all studies based on self-reported behavioral outcomes, the measures are subject to reporting errors and biases.

Given the high levels of recall bias and social desirability bias usually associated with condom use survey items, a measure that identifies a lower proportion of condom users than initially found is taken to be more accurate. The two follow-up questions examined in this paper appear to substantially reduce the proportion of people claiming consistent condom use. As most behavioral surveys rely on self-reported measures, the addition of such questions could significantly improve estimates of consistent condom use. Further research is warranted to refine such follow-up items. For example, asking whether a condom was used from the beginning to end of intercourse is one of multiple ways of assessing correct condom use; it could also be assessed as a measure of condom slippage or breakage during sexual intercourse, or as a measure of the knowledge and skills required for correct condom use. The appropriateness of the type of follow-up question in different settings and with different target groups needs to be investigated further.

Potential variations notwithstanding, our overall conclusion is that in all surveys asking about self-reported condom use, steps must be taken to improve the validity of this measure. This is particularly important for behavioral surveillance surveys and for surveys which seek to evaluate the effect of interventions to increase condom use: trends over time and changes due to interventions may be more difficult to track with inflated measures. We therefore recommend that these and other types of follow-up items be added to future condom use surveys and evaluated further as potential means of obtaining more accurate information about this important behavior.

## Competing interests

The authors declare that they have no competing interests.

## Authors' contributions

VL contributed to the conceptualization of the study, the data analysis, and is the primary writer of the text. KL contributed to the conceptualization of the study, the data analysis, and edited the text. JB contributed to the data analysis and edited the text. All authors read and approved the final manuscript.
